# Wastewater surveillance for bacterial targets: current challenges and future goals

**DOI:** 10.1128/aem.01428-23

**Published:** 2023-12-15

**Authors:** Sarah E. Philo, Kara B. De León, Rachel T. Noble, Nicolette A. Zhou, Rashed Alghafri, Itay Bar-Or, Amanda Darling, Nishita D'Souza, Oumaima Hachimi, Devrim Kaya, Sooyeol Kim, Katrin Gaardbo Kuhn, Blythe A. Layton, Cresten Mansfeldt, Bethany Oceguera, Tyler S. Radniecki, Jeffrey L. Ram, Lauren P. Saunders, Abhilasha Shrestha, Lauren B. Stadler, Joshua A. Steele, Bradley S. Stevenson, Jason R. Vogel, Kyle Bibby, Alexandria B. Boehm, Rolf U. Halden, Jeseth Delgado Vela

**Affiliations:** 1 Department of Civil and Environmental Engineering and Earth Sciences, University of Notre Dame, Notre Dame, Indiana, USA; 2 School of Biological Sciences, University of Oklahoma, Norman, Oklahoma, USA; 3 Department of Earth, Marine, and Environmental Sciences, University of North Carolina at Chapel Hill, Institute of Marine Sciences, Morehead City, North Carolina, USA; 4 Department of Environmental and Occupational Health Sciences, University of Washington, Seattle, Washington, USA; 5 International Center for Forensic Sciences, Dubai Police, Dubai, UAE; 6 Israel Ministry of Health, Jerusalem, Israel; 7 Department of Civil and Environmental Engineering, Virginia Tech, Blacksburg, Virginia, USA; 8 Department of Fisheries and Wildlife, Michigan State University, East Lansing, Michigan, USA; 9 School of Chemical, Biological and Environmental Engineering, Oregon State University, Corvallis, Oregon, USA; 10 School of Public Health, San Diego State University, San Diego, California, USA; 11 Department of Civil and Environmental Engineering, University of California Berkeley, Berkeley, California, USA; 12 Department of Biostatistics and Epidemiology, University of Oklahoma Health Sciences Center, Oklahoma City, Oklahoma, USA; 13 Clean Water Services, Hillsboro, Oregon, USA; 14 Environmental Engineering Program, University of Colorado Boulder, Boulder, Colorado, USA; 15 School of Chemical, Biological and Environmental Engineering, Oregon State University, Corvallis, Oregon, USA; 16 Department of Physiology, Wayne State University School of Medicine, Detroit, Michigan, USA; 17 Ceres Nanosciences, Manassas, Virginia, USA; 18 Environmental and Occupational Health Sciences Division, University of Illinois Chicago School of Public Health, Chicago, Illinois, USA; 19 Civil and Environmental Engineering, Rice University, Houston, Texas, USA; 20 Department of Microbiology, Southern California Coastal Research Project, Costa Mesa, California, USA; 21 Earth and Planetary Science, Northwestern University, Evanston, Illinois, USA; 22 School of Civil Engineering and Environmental Science, University of Oklahoma, Norman, Oklahoma, USA; 23 Department of Civil and Environmental Engineering, Stanford University, Stanford, California, USA; 24 School of Sustainable Engineering and the Built Environment, Arizona State University, Tempe, Arizona, USA; 25 Department of Civil and Environmental Engineering, Duke University, Durham, North Carolina, USA; 26 Department of Civil and Environmental Engineering, Howard University, Washington, District of Columbia, USA; Centers for Disease Control and Prevention, Atlanta, Georgia, USA

**Keywords:** wastewater surveillance, bacteria, wastewater-based epidemiology

## Abstract

Wastewater-based epidemiology (WBE) expanded rapidly in response to the COVID-19 pandemic. As the public health emergency has ended, researchers and practitioners are looking to shift the focus of existing wastewater surveillance programs to other targets, including bacteria. Bacterial targets may pose some unique challenges for WBE applications. To explore the current state of the field, the National Science Foundation-funded Research Coordination Network (RCN) on Wastewater Based Epidemiology for SARS-CoV-2 and Emerging Public Health Threats held a workshop in April 2023 to discuss the challenges and needs for wastewater bacterial surveillance. The targets and methods used in existing programs were diverse, with twelve different targets and nine different methods listed. Discussions during the workshop highlighted the challenges in adapting existing programs and identified research gaps in four key areas: choosing new targets, relating bacterial wastewater data to human disease incidence and prevalence, developing methods, and normalizing results. To help with these challenges and research gaps, the authors identified steps the larger community can take to improve bacteria wastewater surveillance. This includes developing data reporting standards and method optimization and validation for bacterial programs. Additionally, more work is needed to understand shedding patterns for potential bacterial targets to better relate wastewater data to human infections. Wastewater surveillance for bacteria can help provide insight into the underlying prevalence in communities, but much work is needed to establish these methods.

**IMPORTANCE:**

Wastewater surveillance was a useful tool to elucidate the burden and spread of SARS-CoV-2 during the pandemic. Public health officials and researchers are interested in expanding these surveillance programs to include bacterial targets, but many questions remain. The NSF-funded Research Coordination Network for Wastewater Surveillance of SARS-CoV-2 and Emerging Public Health Threats held a workshop to identify barriers and research gaps to implementing bacterial wastewater surveillance programs.

## INTRODUCTION

After SARS-CoV-2 was detected in fecal material and sewage early during the COVID-19 pandemic (1–3), wastewater surveillance of the virus was implemented around the world. As of April 2023, wastewater-based epidemiology (WBE) for SARS-CoV-2 has been conducted globally at more than 4,000 sampling sites in over 70 countries (4). Wastewater surveillance refers to the systematic testing of wastewater to collect health-related data, while WBE refers specifically to relating wastewater data back to the population (5). After seeing the utility of WBE during the pandemic, public health labs and researchers are assessing other public health gaps that might be addressed using WBE. In July 2022, the United States Centers for Disease Control and Prevention (US CDC) announced the National Wastewater Surveillance System (NWSS) will include additional targets such as antimicrobial resistance (AMR) and etiologies of foodborne illness (6). To help with this growth, the National Academies of Sciences, Engineering, and Medicine (NASEM) formed a committee assessing the lessons learned with SARS-CoV-2 wastewater surveillance and envisioning the future of the field. Their initial report defined the criteria for selecting new wastewater surveillance targets: public health significance, analytical feasibility for wastewater surveillance, and usefulness of community-level wastewater data (7). They presented influenza, antimicrobial resistance, and enterovirus D68 as case studies of pathogens that fit these criteria (7).

Although the NASEM report did not name targets directly, bacteria have routinely been discussed within the research community as potential targets (8–11). However, there is not nearly as much research focusing on bacteria. Abundant questions remain about the applicability of existing methods to bacteria and how wastewater data relate to the number of human infections. A systematic review recently identified 100 different manuscripts describing wastewater surveillance programs of 44 different infectious targets (10). Only two of the targets they identified were bacteria: *Escherichia coli* and *Salmonella* species (10). It is clear the research on bacterial wastewater surveillance and WBE is less abundant compared to viral targets. There are established standardized protocols to test surface water for bacterial markers of fecal contamination (12), of which not all are pathogens, the primary target of wastewater surveillance. However, these methods are primarily used to ensure compliance with the Clean Water Act and are not applicable to wastewater surveillance of pathogenic bacteria (13). Furthermore, wastewater is a very different matrix than surface water, with a more diverse microbial community (14) and more molecular inhibitors relative to surface water (15). This suggests method adjustments are likely needed to detect target pathogens.

Because there is little peer-reviewed and published research validating methods for bacterial WBE, many groups are using pre-analytical (i.e., sampling, matrix concentration, and nucleic-acid extraction) methods developed for SARS-CoV-2 surveillance. However, bacteria and viruses have fundamentally different morphology, and their fate and transport in a wastewater system are determined by different factors. According to a review by Chahal et al. (16), bacterial-particle associations are driven mainly by particle size, composition, and growth stage of the bacteria. Conversely, viral-particle associations are primarily driven by the surface charge of the particle and virion and viral morphology (16). This suggests that methods derived for viral surveillance are not universally applicable to bacterial targets. Additionally, it is not expected that human viral pathogens multiply in wastewater, but whether pathogenic bacteria multiply in wastewater or not is unclear.

In response to these concerns, the Wastewater Surveillance for SARS-CoV-2 and Emerging Public Health Threats Research Coordination Network (RCN) hosted a workshop in April 2023 in which all network members were invited to participate. The goals of the workshop were to discuss current bacterial surveillance in wastewater practices and facilitate knowledge sharing, identify challenges and barriers to implementing bacterial surveillance programs, and recognize paths to overcome those barriers. The products of those discussions are presented here to help guide future work for bacterial wastewater surveillance.

## WORKSHOP STRUCTURE

The RCN was launched in August 2020 to advance research for SARS-CoV-2 wastewater surveillance. It evolved in 2022 to include emerging public health threats. In April 2023, the RCN organized a virtual workshop to discuss existing bacterial wastewater surveillance programs and challenges to implementing new programs. The workshop was open to anyone on the RCN email list and their colleagues but required registration, which included a pre-workshop survey. The workshop had participants at all stages of their career, from graduate students to late career professionals. Participants also represented diverse fields and industries, with attendees from universities, water utilities, government agencies (including public health authorities), and private businesses. The first part of the workshop involved a panel with presentations about existing bacterial monitoring programs. Topics discussed included bacterial partitioning in wastewater compared to viral partitioning and surveillance for AMR and *Salmonella enterica* serovar Typhi (*S*. Typhi). Rather than providing specific guidance, panelists Kara B. De León, Rachel T. Noble, and Nicolette A. Zhou were asked to talk about their own research experiences with the goal of encouraging conversation among workshop participants.

De León discussed modifying existing methods for viral nucleic acid extractions from wastewater to co-extract bacterial targets. Compatibility issues could arise with existing methods. For example, solid removal steps may also remove bacterial cells. In addition, there are losses in efficiency when isolating high and low molecular weight nucleic acids simultaneously. Targets for bacterial pathogen quantification are often located on the chromosome rather than on a plasmid, but many pathogenic and resistance genes are located on plasmids (17). Estimating cell numbers from gene copies relies on the assumption that there is one gene target per bacterial cell, but plasmid copy number per cell ranges by orders of magnitude depending on the plasmid (18). Therefore, gene targets on plasmids may not accurately estimate bacterial concentrations. Additionally, bacterial chromosomes are much larger than the genomes of viral targets. Methods that have been optimized for extracting small viral nucleic acids may not also efficiently extract larger bacterial chromosomal DNA. The loss of this bacterial target signal, whether by cell removal or inefficient nucleic acid extraction would lead to an underestimation of bacterial pathogen concentrations in the wastewater. Furthermore, concentration of bacterial cells with centrifugation can also concentrate inhibitors. Additional steps may be required to remove inhibitors while retaining the bacterial fraction. While not impossible to co-concentrate and extract viral and bacterial nucleic acids, labs will be required to critically assess methods to control for these challenges.

Noble’s talk focused on wastewater surveillance for AMR and pathogenic bacteria. She started by discussing the utility of both quantitative PCR (qPCR) and digital PCR (dPCR) in wastewater surveillance. While dPCR has become the standard for COVID-19 surveillance, qPCR can be easily multiplexed and is more adaptive to new targets such as AMR. Noble additionally discussed adapting methods to bacterial targets. Viral genes targeted with PCR methods are species specific, and detection of the gene confirms the presence of the virus. While antimicrobial resistance genes (ARGs) can move between species and proliferate in the environment in pathogenic and non-pathogenic bacteria in wastewater, biofilms, and soils, suggesting detection of the gene does not confirm the presence of a specific bacterial species. Additionally, when conducting surveillance for bacteria, it is crucial to confirm the presence of both the species and the pathogenic genes to better understand underlying infections. Because many human pathogens are viable but non-culturable (VBNC) (19), it is difficult to definitively link pathogenic genes to their host species because this is commonly carried out with culture.

Zhou focused on concentration methods, use cases, and challenges of environmental surveillance of *S*. Typhi (20). Trap and grab sampling methods are used globally for *S*. Typhi, and the choice depends on the use case scenario (vaccine campaign location selection, vaccine campaign monitoring, low concentration detection/early outbreak detection, and disease surveillance). There are many challenges with culture- and molecular-based *S*. Typhi detection methods. For culture-based methods, there are competing organisms and some of the media used is inhibitory and hazardous, leading to safety and disposal concerns. For molecular methods, the widely used qPCR assay was developed using clinical samples and the targets are not fully specific to *S*. Typhi (21), making it challenging to confirm detection in wastewater using qPCR alone. Inclusion of multiple pan-*Salmonella* and *S*. Typhi targets facilitates detection in wastewater (22), and confirmation can be accomplished through sequencing. Finally, molecular detection with environmental samples can be challenging due to low *S*. Typhi concentrations and the complex background matrix.

After the panel, participants were then put into breakout rooms. They were asked to discuss the targets, methods used, and goals of existing bacterial wastewater surveillance programs, and challenges or barriers they faced and what information is needed to overcome those barriers. After the breakout rooms, the groups reported a few of the main points of the smaller room’s discussion with the whole workshop. After the workshop, notes in the workshop Google document were compiled by the organizers to identify what work is being done and highlight areas where more information is needed to implement bacterial monitoring programs as identified by workshop participants (Google Inc., Mountain View, CA, USA). RStudio and its associated packages were used to collate data and develop the figures (2019 RStudio: Integrated Development for R. Version 1.2.5033 RStudio, Inc., Boston, MA, USA, URL http://www.rstudio.com/).

## EXISTING BACTERIAL MONITORING PROGRAMS

**Fig 1:**
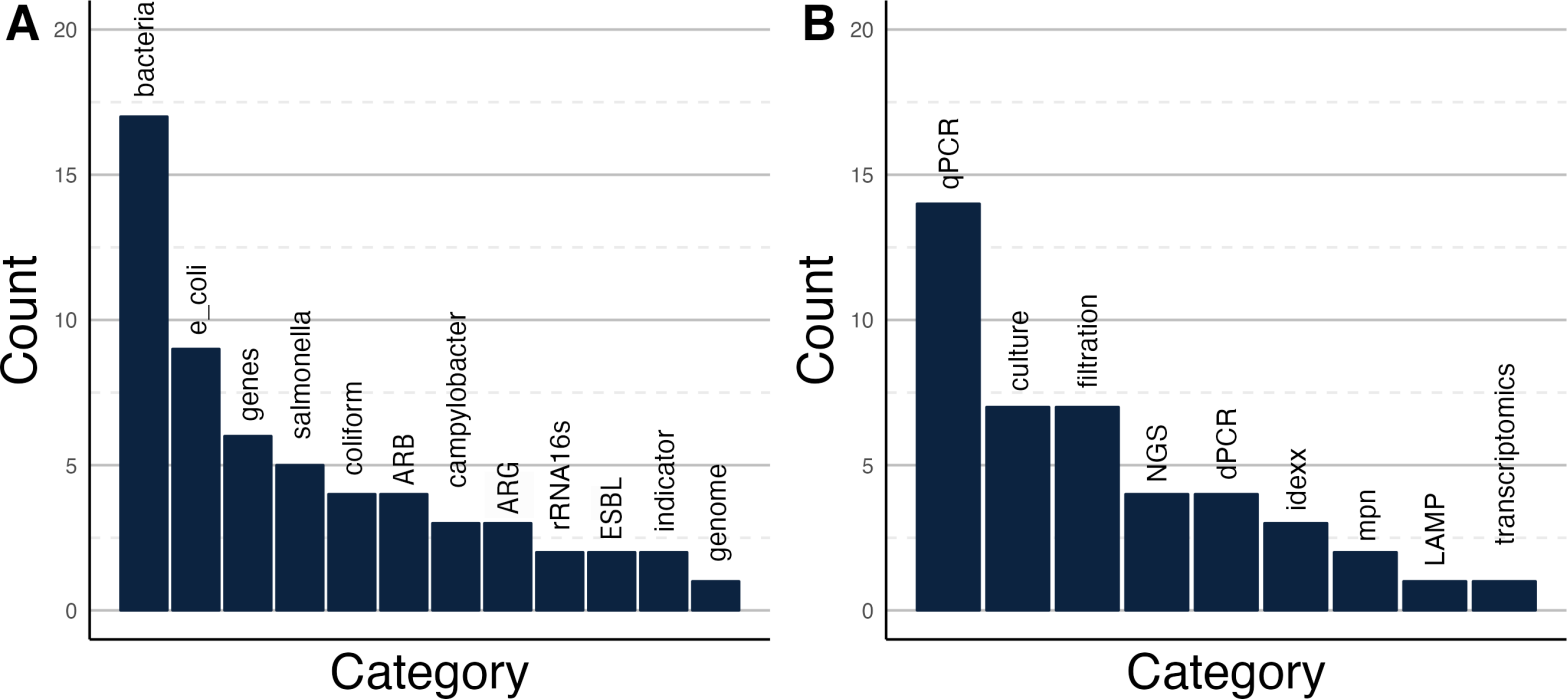
Author responses collected before the workshop on (A) the types of bacterial targets and (B) the methods used in wastewater surveillance programs. Twelve different targets (**A**) and nine different methods (**B**) were discussed. This indicates there is substantial diversity in bacterial targets and methods being used in wastewater surveillance programs. Acronyms: (A) ARB – antimicrobial resistant bacteria; ARG, antimicrobial resistance gene; ESBL, extended spectrum beta lactamase (B) NGS, next-generation sequencing; LAMP, loop-mediated isothermal amplification.

Participants were asked to discuss their experience with existing or previous bacterial wastewater surveillance programs. Despite a general lack of standardized approaches for bacterial wastewater monitoring, the targets discussed were incredibly diverse, with 16 different targets mentioned in breakout room discussions, highlighting increased interest (Table 1). Of note, it is unclear if discussions focused specifically on programs using wastewater to understand human disease prevalence or programs that are attempting to characterize bacteria generally in the environment. While most of the targets discussed are fecal associated bacteria, other targets, such as *Legionella*, *Klebsiella*, and *Pseudomonas* genera, cause respiratory infections. Numerous people discussed surveillance programs for AMR, including twelve different resistance genes and four different resistant bacteria. Responses collected individually before the workshop further support the diversity of bacterial targets in monitoring programs (Fig. 1A). Twelve different targets were mentioned in responses collected before the workshop, indicating there is much diversity in interest for bacterial wastewater monitoring. However, it is again unclear if targets were mentioned for the specific purpose of using wastewater data to better understand disease prevalence and incidence, or for generally describing bacterial communities in the environment.

**TABLE 1:** Bacterial targets for wastewater surveillance discussed during breakout room sessions^*c*^

Target	Times mentioned
Antibacterial resistance genes^*a*^	4
Antimicrobial resistant organisms^*b*^	4
*Salmonella* spp.	4
*Campylobacter* spp.	3
*Escherichia coli*	3
Shiga toxin-producing *E. coli*	2
*Vibrio* spp.	2
*Klebsiella* spp.	1
*Listeria* spp.	1
Fecal indicator bacteria	1
Human fecal markers	1
*Legionella* spp.	1
*Pseudomonas* spp.	1
*Bacterioides* HF183	1
*Dehalococcoides*	1
Reductive dechlorination biomarkers	1

^*a*^ Genes discussed: sul1, intl1, tetA, ermB, qnrS, VanA, blaTEM, blaCTX, blaCMY, mecA, mcr1, blaSHV.

^*b*^ Organisms discussed: Extended-spectrum beta-lactamase resistance, Carbapenem resistant *Enterobacterales*, Vancomycin resistant *Enterobacterales*, Methicillin resistant *Staphylococcus aureus.*

^*c*^ Notes taken on the provided Google document during discussions were analyzed to pull bacterial target information from the conversations. Sixteen different targets were discussed, with only seven being mentioned multiple times.

Breakout room discussions identified diverse methods in use to detect bacteria in wastewater (Table 2). Molecular techniques such as dPCR and qPCR were the most discussed in the breakout rooms. The most mentioned method in individual responses collected before the workshop was qPCR (Fig. 1B). Multiple different concentration methods were discussed, with various IDEXX and most-probable number (MPN) methods mentioned both in individual responses and in break out rooms (Table 2; Fig. 1B). There were nine different methods mentioned in responses collected before the workshop, suggesting methods being used by research groups are diverse. The diversity in methods suggested is likely a product of the highly diverse targets provided in Table 1, as some nonculturable organisms would only be detectable with molecular methods. As discussed in the panel discussion, concentration and extraction methods should be optimized for the target of interest.

**TABLE 2:** Methods used for wastewater surveillance discussed during breakout room sessions^*a*^

Method	Times mentioned	Notes
dPCR	6	
qPCR	4	
Culture	3	Culturing to confirm molecular results mentioned twice
Metagenomics	3	
Flocculation	2	Skimmed milk flocculation mentioned once
Filtration	2	Hollow fiber ultrafiltration mentioned once
Extraction	2	Solid/liquid phase, magnetic beads mentioned once each
Colilert	1	
Amplicon Sequencing	1	
Nanotrap Magnetic Beads	1	

^*a*^ Information was pulled from notes taken on the document, with ten different methods being mentioned. Only three were mentioned once.

The goals of existing surveillance programs were also discussed (Table 3). Source tracking, water monitoring, and disease and outbreak surveillance were most frequently mentioned. Microbial source tracking (MST) is one of the better described and standardized water monitoring techniques. The ultimate goal of MST is to identify sources of fecal contamination to manage surface water quality (23). Molecular markers have historically been the most commonly used detection method for MST (23). The EPA has various standardized protocols to monitor water for fecal contamination (12). Understandably, these were frequently identified as the goal of existing programs of workshop participants. Many of the goals discussed would not be considered WBE or wastewater surveillance for public health benefits but focus on the more traditional uses of bacterial monitoring for research and tracking fecal contamination.

**TABLE 3:** Goals of existing bacterial wastewater monitoring programs^*a*^

Goal	Times mentioned
Disease/outbreak surveillance	5
Source Tracking	3
Water monitoring	2
Method validation/pilot tests	2
Horizontal gene transfer	1
Bioremediation	1
Trend Surveillance	1
Vaccine development	1
Informing public agencies	1
Wastewater-based epidemiology	1
Sewage treatment	1
Finding uses for bacterial targets	1

^*a*^ Participants discussed how bacterial monitoring programs are being used currently. Many of the goals do not fall under the definition of wastewater surveillance but focus more on environmental engineering/health aims or general research.

## CHALLENGES AND BARRIERS TO BACTERIAL WASTEWATER MONITORING

The participants were also asked to discuss specific challenges and barriers they have faced in implementing bacterial wastewater surveillance programs. The concerns fell into four main categories: (1) prioritizing new targets, (2) relating lab results to human infections, (3) choosing methods, and (4) normalizing results. There were also limited discussions about the ethics of bacterial wastewater surveillance, environmental factors that might affect wastewater surveillance, and how to interpret viable but non-culturable (VNBC) results.

### Choosing new targets

There were discussions about how to choose bacterial targets to include in wastewater surveillance programs, with public health utility a critical focus. The discussions generally focused on both feasibility and priority. Organisms that have a high priority for public health disease surveillance may not be suitable for WBE (e.g., not detected in feces or urine) or take too long to receive actionable results due to methods limitations. It is crucial that specific communication and action plans be decided before implementing wastewater surveillance programs. Whether quantitative data are needed to assess disease trends or presence/absence data are needed to detect emergence into a community must be decided first. Designing wastewater surveillance programs moving forward will require extensive communication between researchers and public health practitioners so that wastewater surveillance can successfully complement traditional disease surveillance programs.

There are a few proposed methodologies for choosing new pathogens for wastewater surveillance. The NASEM recently convened a committee that discussed this topic (7). They identified three things to consider for identifying candidate pathogens. First, what is the public health significance of the threat? Second, is it analytically feasible to conduct wastewater surveillance for this target? Third, will the data be useful to inform specific public health actions? An additional ranking system was recently published by Gentry et al. (24). This considers reportable diseases, transmissibility, local case rates for the Detroit-area, and analytical feasibility to produce a numeric score (24). Diseases with higher scores should have higher priority. However, this was developed specifically for the Detroit-area. Moving forward, increasing adoption of both the NASEM criteria and ranking systems like the one produced by Gentry et al. (24) will allow for improved resource allocation toward infectious disease targets of public health value. Research efforts to optimize sampling, concentration, and extraction methods should be focused on these targets to ensure successful implementation by public health professionals.

### Relation between wastewater data and human infections

The authors believe some of the largest research gaps deal with relating bacterial wastewater data to human infections. While routine monitoring of SARS-CoV-2 has revealed well-established relationships between wastewater and clinical data for a number of monitoring programs (25–27), this relationship, if it exists, is not well studied for bacteria. More research is needed to establish the shedding patterns of potential bacterial targets. Additionally, it is frequently difficult to understand if detection is due to commensal or pathogenic strains. There is also limited research on bacterial dynamics in the wastewater environment and sewer network. Participants had questions about whether target bacteria multiplied in the sewer system or primarily decayed due to death or adsorption. Additionally, given that many human bacteria have similar environmental or animal strains, methods are often not specific enough to distinguish between these types. For example, some methods will give the user bacterial concentration as an MPN (28), but not necessarily where those bacteria came from originally or whether their genomes include toxin genes or other markers of pathogenicity. The possibility that assays may not distinguish between environmental and human bacteria necessitates the development of background thresholds for certain targets, but again this is not well established.

The final problem with relating wastewater data to human infections involves the widespread use of metagenomic sequencing to detect bacterial communities or pathogenic elements. It is often difficult to definitively link specific ARGs or pathogenic genes to bacterial taxa with shotgun sequencing. Targeted sequencing panels, such as the Respiratory Pathogen ID/AMR Enrichment Panel (Illumina, San Diego, CA, USA), have been used to look for specific ARGs and bacteria in wastewater (29), but they still cannot definitively link genes to host taxa. Long-read sequencing has been shown to better link ARGs to their hosts (30), but plasmid-borne ARGs are still difficult to link to their hosts with long-read sequencing. While single-cell sequencing protocols, such as epicPCR and Hi-C (31, 32), have successfully linked ARGs to their hosts, they are time consuming and resource intensive. Furthermore, the detection of a particular gene does not mean that gene is expressed by the bacteria. The gold standard for determining the expression of a resistance gene, for example, requires isolating and culturing individual colonies of bacteria in the presence of selective antibiotic media (33, 34). However, a vast majority of bacteria are not culturable (35), limiting the applicability of culturing for wastewater surveillance.

### Choosing a method

Workshop participants identified an abundance of factors that must be considered when designing a sampling and analysis protocol for bacterial surveillance programs. There were discussions around whether concentration of the sampled matrix is needed to increase sensitivity for certain targets. Wastewater concentration is often necessary to detect rare targets. However, methods used to concentrate wastewater can also concentrate molecular method inhibitors (36). Tradeoffs between assaying large volumes of wastewater to search for rare targets and subsequent inhibition in detection assays must be carefully evaluated. Additionally, the sensitivity of existing methods for bacterial wastewater surveillance is not well discussed in the literature. Understanding the limits of detection is crucial to choosing a method. Whether to prioritize highly sensitive methods or methods less affected by inhibitors will be dependent on the target and the surveillance program goal. The sampling location and frequency must be considered for each application as certain schemas are more appropriate than others. More discussion about specific use cases is needed to implement a sampling and wastewater concentration plan.

There are additional questions about how and when to use culture-based methods, in addition to molecular detection, and whether enrichment is needed. Molecular results do not always agree with cultured results, particularly for fecal indicator bacteria (37, 38). While the EPA protocols for measuring bacteria in surface waters or biosolids require culturing for specific bacteria with selective media (12, 39), these culture methods may not be sensitive enough to distinguish between pathogenic bacteria, and may also take weeks to receive results. Participants also discussed culturing bacteria to confirm molecular results, and vice versa, but there is little consensus on when these steps are needed. Additionally, because it is impossible to confirm molecular results with culture for VBNC bacteria, a negative culture result will lead to false negatives in the overall data.

To help with data interpretation, the participants discussed the need for a best practices document for discussing and reporting methods. There are a few existing resources for this information. The US CDC lists metadata required to interpret wastewater measurements on the NWSS website (40), and the Environmental Microbiology Minimum Information (EMMI) Guidelines include a checklist to ensure that necessary information are listed in the publication (41). However, neither of these resources was developed specifically for bacterial wastewater surveillance. Further assessment as to how these may apply for bacterial surveillance is necessary.

### Result normalization and controls

Normalization targets are widely used to control for the amount of human fecal matter present in the wastewater. These include flow rate, viral targets such as PMMoV, crAssphage, and F + coliphage, and the bacterial target HF183 Bacteroides (42, 43). The US CDC also lists non-biological targets such as caffeine, creatinine, and ibuprofen as potential normalization controls (40). Except for HF183, these are either non-biological or viral targets. How well they function to normalize bacterial wastewater surveillance data is yet to be thoroughly assessed. Importantly, the choice of normalization target will likely change depending on the type of bacteria the researcher is looking for and the goal of normalization, as Gram-positive and Gram-negative bacteria have different surface chemistries and possibly partition differently in wastewater. Because enveloped and non-enveloped viruses may partition differently between the solid and liquid fraction in wastewater (44, 45), the same thorough assessment is needed to understand how bacteria partition differentially. This will affect the methods chosen as it will determine which fraction of wastewater to focus concentration efforts. There are also gaps in the types of organisms that should be used for recovery, process, and inhibition controls. The EMMI guidelines describe in detail when each of these controls should be used and provide a framework for distinguishing between controls used at different steps of the process (41). It also provides proposed definitions and vocabulary that should be carried forward with bacterial wastewater surveillance to reduce confusion.

## A PATH FORWARD

The workshop participants identified a number of steps to help facilitate wastewater surveillance for bacterial targets. First, a document outlining data-reporting standards and necessary quality control steps must be developed. The RCN previously published a standardized data-reporting document (46). A similar document should be developed for bacterial wastewater surveillance. This will require method validation and optimization on seeded samples in laboratories to understand the metadata needed to interpret results. Future discussions are also needed to identify and validate normalization markers and spike-in controls for bacterial monitoring.

Given the difficulty in linking ARGs with their host bacteria, more efficient and less resource intensive methods must be developed. While it is possible to detect ARGs and their host bacteria simultaneously with methods such as Hi-C and epicPCR (30–32), they are expensive and require substantial lab resources. Additionally, studies must be conducted to improve our understanding of understand the clinical relevance of molecular ARG detection. Long-read sequencing has the potential to determine whether ARGs are in clinically relevant bacteria but provides little information about whether they can be turned on or can move easily between organisms. Better phenotypic expression studies are needed to understand these characteristic relationships.

More research is also needed to describe shedding patterns for both symptomatic and asymptomatic infections to develop specific shedding models to better compare wastewater and clinical data. The authors call on our clinical colleagues to conduct studies assessing shedding for bacterial pathogens in their patients and reporting the results in a way that is congruent with wastewater surveillance activities. Data metrics such as gene copies or cells per gram of feces would allow for greater applicability to WBE. This is particularly important for antimicrobial resistant bacteria (ARB). While numerous studies have described ARB shedding in dairy cattle (47), comparatively less research has studied ARB shedding quantitatively in humans. Additionally, clinical fecal shedding studies are often carried out to help characterize symptoms and improve disease management, but these data are not always reported in a way that is useable for wastewater surveillance. Arts et al. (48) present quantitative shedding data for PMMoV and crAssphage on an individual level to aid in WBE applications. Systematic reviews of existing research to build shedding models will help the interpretation of bacteria wastewater surveillance results. Ultimately, wastewater surveillance requires the breaking down of traditional academic and clinical research silos to better understand disease spread.

The authors also feel greater focus on the ethics of wastewater surveillance is needed. As wastewater surveillance transitions away from being used in an emergency setting, ethical best practices need to be built in at every step of the process. There have been prior discussions about some of the ethical considerations for infectious disease surveillance (49–51). Additionally, a guidance document for wastewater surveillance of illicit drugs was developed in 2016 by the European Monitoring Centre for Drugs and Drug Addiction (52). However, no such document exists for bacterial wastewater surveillance. Public Health Canada developed an ethical guidance document for SARS-CoV-2 wastewater surveillance (53), but the applicability of this document to non-pandemic bacterial targets is unclear. The authors recommend scoping principles be established to ensure programs moving forward have guidance to inform their ethical practice for wastewater surveillance of targets beyond SARS-CoV-2.

Wastewater surveillance has the potential to give public health officials and clinicians greater insight into the true disease burden in their communities. However, much work remains to be done as wastewater surveillance expands beyond the COVID-19 pandemic. This document sheds light on areas for improvement and can help guide future research in this field.

## AFTER EPUB

*[This article was published on 15 December 2023 with a byline that lacked Jason R. Vogel. The byline was updated in the current version, posted on 24 January 2023.]*
